# P02.48. Overcoming ego depletion: the effects of an optimism manipulation on repeated acts of self-control

**DOI:** 10.1186/1472-6882-12-S1-P104

**Published:** 2012-06-12

**Authors:** Y Meevissen, M Peters, H Alberts

**Affiliations:** 1 Maastricht University, Maastricht, Netherlands

## Purpose

The present study attempted to establish the causal status between optimism and executive functioning (self-regulation). Repeated acts of self-control draw from a limited general resource which, upon depletion, results in reduced capacity for continued self-regulation, a process termed ego depletion (Baumeister, Bratslavsky, Muraven, & Tice, 1998). The present study investigated whether: (1) An *a priori* explicit optimism induction can buffer against the ego depletion effect. (2) A potential reduction in the ego depletion effect caused by optimism results from (a) more effective mental resource utilization or (b) greater willingness to invest resources.

## Methods

Eighty female participants were randomly assigned to a 2 (optimism vs. neutral) x 2 (depletion vs. no-depletion) factorial between-subjects design. For 20 minutes, participants were either instructed to describe and visualize their best possible self (BPS) (optimism condition) or their daily rituals (DR) (neutral condition). After this manipulation half of the participants were depleted by performing a difficult Stroop task. The other half remained non-depleted by performing an easy Stroop task. Following the Stroop task, all participants performed two other self-control demanding tasks. As a measure of self-control performance, participants were requested to pinch a handgrip as long as possible on various time points during the lab session.

## Results

Visualizing a best possible self was shown to successfully enhance optimism. Whereas participants in the control condition demonstrated the effect of ego depletion (expressed by reduced self-control performance), participants in the optimism condition did not suffer from this decrease in self-control performance, not even after carrying out repeated acts of self-control.

## Conclusion

Optimism seems to be able to buffer against the effects of ego depletion, thereby enabling people to carry out repeated acts of self-control, which has for instance been indicated to contribute to goal pursuit, goal achievement, and resilience in times of adversity or stress.

